# Boosted lopinavir vs boosted atazanavir in patients failing a NNRTI first line regimen in an urban clinic in Kampala

**DOI:** 10.7448/IAS.17.4.19792

**Published:** 2014-11-02

**Authors:** Eva Laker, Ivan Mambule, Damalie Nalwanga, Joseph Musaazi, Agnes Kiragga, Rosalind Parkes-Ratanshi

**Affiliations:** 1 Prevention, Care and Therapy, Infectious Diseases Institute, Kampala, Uganda; 2 Training and Capacity Building, Infectious Diseases Institute, Kampala, Uganda; 3 Research, Infectious Diseases Institute, Kampala, Uganda

## Abstract

**Introduction:**

In 2011 Uganda recommended boosted atazanavir (ATV/r) as the preferred PI for second line due to once daily dosing, replacing aluvia (LPV/r) [1, 2]. The evidence was based on the BMS O45 trial, of LPV/r vs ATV/r was performed in a high-income setting, on patients with prior PI use and resistance testing [2, 3]. There are no RCTs or observational studies comparing use of ATV/r with LPV/r in patients failing NNRTI first line antiretroviral therapy in sub-Saharan Africa [3, 4]. The Infectious Diseases Institute (IDI) has a large second line cohort (>1838). This aims to compare clinical, immunologic and virologic response of LPV/r versus ATV/r at IDI.

**Methods:**

Retrospective cohort analysis on routinely collected data of patients switched to second line with NRTI backbones TDF/3TC or FTC, AZT/3TC, ABC/3TC from January 2009 to December 2013. Students T-tests and Chi-square tests were used in this analysis.

**Results:**

A total of 1286 (73.5% female) patients were switched to LPV/r 991 (77%) and ATV/r 295 (23%) (p<0.001). NRTI backbones were 760 on TDF/3TC (66.8% LPV/r vs 33.2% on ATV/r), 504 on AZT/3TC (93.3% vs 6.7%), and 22 on ABC/3TC (59% vs 41%). Median (IQR) time on first line for LPV/r was 21 (1–44) months and for ATV/r was 41 months (22–68). Median CD4 (IQR) at switch to LPV/r was 181 cells/uL (66–424) and to ATV/r was 122 (57–238) (p≤0.001). A total of 366 patients had CD4 done at six months after switch and the mean (IQR) CD4 increase was 153 (54–241) for LPV/r versus 116 (52–171) for ATV/r (p=0.232). Additionally, 304 had a CD4 at 12 months and the means were 172 (45–272) for LPV/r vs 179 (60–271) for ATV/r (p=0.426). There was no significant difference in the mean increment by NRTI backbone or by stratifying to viral load (VL) at time of switch to VL <100,000 and ≥100,000. Median (IQR) VL at switch was 61,000 (13,000–2,030,000) LPV/r and 51,000 (14,000_151,000) ATV/r. 269 had a VL done in the first 12 months and 178/250 (71.2%) on LPV/r versus 16/19 (84.2%) on ATV/r were undetectable (p=0.228). 259 (26%) LPV/r versus 33(11%) ATV/r had ≥1 opportunistic infections on second line (p<0.001).

**Conclusions:**

This is an observational study based on our experience at IDI. Like elsewhere in Africa, there is no routine viral load testing, making it difficult to get sensitive analysis of data on ART efficacy within routine clinical practice. Nevertheless, this observational study is reassuring in terms of efficacy of both ATV/r and LPV/r for patients failing first line therapy in our setting.
